# Gen-2 Hand-Held Optical Imager towards Cancer Imaging: Reflectance and Transillumination Phantom Studies

**DOI:** 10.3390/s120201885

**Published:** 2012-02-10

**Authors:** Jean Gonzalez, Manuela Roman, Michael Hall, Anuradha Godavarty

**Affiliations:** Optical Imaging Laboratory, Department of Biomedical Engineering, Florida International University, 10555 W. Flagler St., Miami, FL 33174, USA; E-Mails: jgonz007@fiu.edu (J.G.); mroma014@fiu.edu (M.R.); mhall003@fiu.edu (M.H.)

**Keywords:** near-infrared, diffuse optical imaging, hand-held imager, hand-held probe, reflectance, transillumination

## Abstract

Hand-held near-infrared (NIR) optical imagers are developed by various researchers towards non-invasive clinical breast imaging. Unlike these existing imagers that can perform only reflectance imaging, a generation-2 (Gen-2) hand-held optical imager has been recently developed to perform both reflectance and transillumination imaging. The unique forked design of the hand-held probe head(s) allows for reflectance imaging (as in ultrasound) and transillumination or compressed imaging (as in X-ray mammography). Phantom studies were performed to demonstrate two-dimensional (2D) target detection via reflectance and transillumination imaging at various target depths (1–5 cm deep) and using simultaneous multiple point illumination approach. It was observed that 0.45 cc targets were detected up to 5 cm deep during transillumination, but limited to 2.5 cm deep during reflectance imaging. Additionally, implementing appropriate data post-processing techniques along with a polynomial fitting approach, to plot 2D surface contours of the detected signal, yields distinct target detectability and localization. The ability of the gen-2 imager to perform both reflectance and transillumination imaging allows its direct comparison to ultrasound and X-ray mammography results, respectively, in future clinical breast imaging studies.

## 1. Introduction

Diagnostic imaging of early-stage breast cancer is essential for decreasing the cancer death rate in the United States. The conventional anatomical based screening techniques (*i.e.*, X-ray) are neither comprehensive nor infallible, especially in women with dense breast tissues. Over the past three decades, near-infrared (NIR) optical imaging approaches have been developed for breast cancer screening based upon the endogenous absorption contrast owing to the non-specific process of angiogenesis, in order to discriminate normal from diseased tissues. NIR light between the wavelengths of 700–900 nm propagates deeply through tissues and provides a unique approach for molecularly-based diagnostic imaging. While there are a number of clinically applied imaging modalities (e.g., X-ray, nuclear, magnetic resonance imaging (MRI), ultrasound), NIR optical techniques can be used to provide complementary functional information without the use of additional harmful radiation (as in X-ray), radio-active substances (as in nuclear), or bulky instrumentation (as in MRI).

In recent years, hand-held based NIR optical imagers are developed in an attempt to translate the near-infrared (NIR) imaging technology to clinic breast imaging [1–22], with maximum patient comfort and portability (against bed-based optical imagers). In general, the hand-held imagers launch and collect the NIR signals from the tissue surface via reflectance imaging using a single (optical fiber based) hand-held probe. These hand-held imagers have been used predominantly for spectroscopic analysis and two-dimensional (2D) tumor detections.

All the hand-held imagers available to date have only flat probe heads, which limit them from: (i) contouring to tissue curvatures with good surface contact; and (ii) obtaining depth information, due to lack of transillumination measurements (since they obtain only reflectance measurements). In addition, it is not appropriate to compare reflected optical images to the gold-standard X-ray mammography images that acquire transilluminated (or transmitted) signals.

A (generation-1 or Gen-1) novel hand-held probe based optical imager was developed in our Optical Imaging Laboratory and its feasibility towards two-dimensional (2D) surface mapping and three-dimensional (3D) tomographic imaging has been demonstrated on cubical tissue phantoms [23–25]. Although the hand-held probe could contour to different tissue curvatures unlike existing hand-held probes, the design was bulky, non-patient comfortable, limited to reflectance measurements (and not transillumination a.k.a. transmittance), and lacked high sensitivity. Hence, a generation-2 (Gen-2) hand-held imager that overcame the above limitations was recently developed [26]. The unique features of the Gen-2 hand-held optical imager include: (i) A flexible probe head that allows the probe to contour along tissue curvatures with maximum contact (∼86%); (ii) A forked probe design that allows both sequential and/or simultaneous bilateral reflectance imaging as well as transillumination (or compressed) imaging, as in ultrasound and X-ray mammography, respectively; and (iii) Multiple sources and detectors that allow sequential and/or simultaneous illumination and simultaneous detection towards rapid imaging of large tissue surfaces (∼20 cm^2^).

The focus of the current work is to demonstrate the feasibility of performing both reflectance and transillumination imaging using this Gen-2 hand-held optical imager. The improvement in deep target detection using transillumination measurements over reflectance is assessed. In addition, new data post-processing techniques have been developed to improve target detectability and from surface optical measurements (both reflectance and transillumination).

## 2. Materials and Methods

### 2.1. Instrumentation

**Figure 1.**
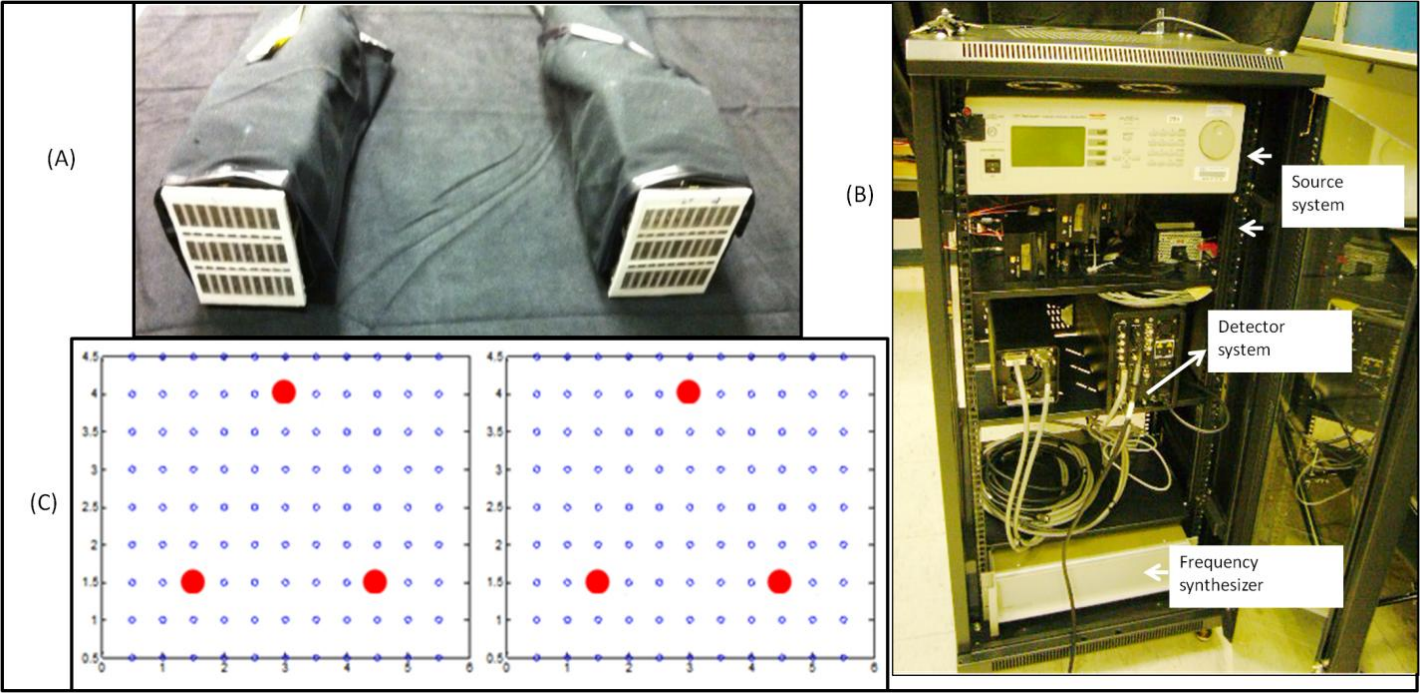
The hand-held optical imager set-up with (**A**) a forked hand-held probe, (**B**) a portable cart containing the source (laser diodes), detector (ICCD camera) and other components, and (**C**) source-detector layout for both probe heads. The red dots represent (sequential or simultaneous) illumination points and the black open circles represent detection points.

The Gen-2 optical imager primarily consists of 3 components; the forked hand-held probe, the illumination system, and the detection system (see Figure 1). The hand-held probe of the Gen-2 optical imager is a forked probe design capable of conforming to tissue surface areas with minimal compression (see Figure 1(A)). The forked probe design allows for both reflectance as well as transillumination imaging approach, unlike all other hand-held imaging systems that are capable of only reflectance imaging [1–22]. There are 3 sources on each of the forked probe head, configured such that there is maximum area of illumination of the imaged region by the 4 × 5 cm^2^ probe head [26]. The 3 sources and 96 detectors are spaced 0.5 cm apart on each probe head (as shown in Figure 1(C)).

The illumination system allows for both sequential as well as simultaneous illumination of the 3 + 3 sources via 6 laser diodes operated using a multi-channel laser diode controller [26]. Currently, the sources provide an optical output power of ∼5mW from each illumination point as a continuous-wave (CW) signal at 785 nm wavelength. Tissue phantoms are illuminated at single or multiple points via the forked probe heads and the detected optical signals is collected via the probe heads’ detection fibers and imaged using an intensified charge coupled device, ICCD camera (PI-MAX2, (Princeton Instruments, Newton, NJ, USA). Neutral density filters are used appropriately to collect the attenuated optical signal at 785 nm wavelength. The ICCD camera’s exposure time and gain settings are adjusted prior to each experimental study, in order to maximize the intensity of the detected signal. The illumination and detection system of the imager are controlled via custom-developed Labview software. The entire instrumentation is assembled onto a portable cart for ease of mobility during our clinical translational efforts (see Figure 1(B)). Extensive details of the instrumentation are provided elsewhere [26].

For the current studies, CW-based imaging was performed on tissue phantoms, where the exposure time of the ICCD camera was maintained at 0.2 s for all the studies. Ten repeated images were acquired at each location during experiments, and the average optical signal was used for further analysis.

### 2.2. Experimental Studies

**Figure 2.**
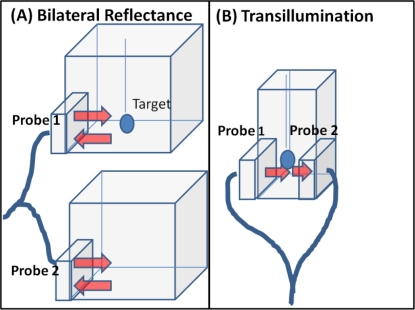
Schematic of experimental tissue phantom study set-up for (**A**) simultaneous bilateral reflectance imaging, and (**B**) transillumination imaging.

Diffuse optical imaging studies were performed on cubical and slab phantoms filled with 3% Liposyn solution (Hospira, Inc. Lake Forest, IL, USA). Spherical targets of 0.45 cc (0.95 cm diameter) filled with 0.08% (by volume) India Ink (absorbing agent) (Chartpak, Leeds, MA.) and 3% Liposyn solution were employed. Reflectance (simultaneous bilateral) and transillumination experiments were performed under different experimental conditions of target depth (1–5 cm), and India Ink target: background (T:B) contrast ratio (e.g., 0.08%:0, and 0.08%:0.0008% or 1:0 and 100:1, respectively). The details of the experimental cases are given in Table 1. During simultaneous bilateral reflectance imaging studies, two 10 × 10 × 10 cm^3^ cubical phantoms were used, one with a target and the other without. All the six point sources on both the probe heads are simultaneously illuminated. During transillumination imaging studies, 5.5 × 5.5 × 10 cm^3^ slab phantoms were used. All the 3 point sources of one probe head are simultaneous illuminated and the point sources on the other probe head (located on the opposite surface of the imaging plane) are not (see Figure 2(B)). In both reflectance and transillumination imaging studies, the target depth is measured w.r.t. the phantom surface that is illuminated (via all simultaneous point sources) by the probe head. These studies will determine if transilluminated measurements detect deeper targets (from 2D surface images) over reflected measurements, upon using a unique hand-held probe capable of acquiring both these measurement types.

The optical properties of 3% Liposyn solution in terms of absorption (μ_a_) and reduced scattering (μ_s_') coefficient are 0.09 and 9.5 cm^−1^, respectively, as measured using our single pixel homodyne optical imaging system. The typical optical properties of human breast tissues are μ_a_ = 0.04 ± 0.02 cm^−1^, μ_s_′ = 8 ± 4 cm^−1^ [27]. Hence, the phantoms used in the current studies have similar optical properties of a human breast tissue. The absorption coefficient of 0.08% India Ink is ∼0.3 cm^−1^ as estimated by researchers in the past (at 750 nm wavelength) [28]. The reduced scattering coefficient (μ_s_′) of India Ink can vary between 4 to 20 cm^−1^ as reported by various researchers [28,29], and was challenging to estimate in our studies.

### 2.3. Data Analysis

The optical data acquired by the ICCD camera is in terms of a.b.u., which corresponds to the detected intensity signal from the various detection points on the imaged surface. These pixelated optical data acquired for the entire imaged region of the ICCD camera is then used to extract meaningful data corresponding to the detection fiber regions (*i.e.*, for 96 × 2 detectors). The extracted optical data (or intensity data) corresponding to the 96 detected points of each probe head on the imaging surface is averaged across the 10 repeated measurements. The averaged intensity data corresponding to the 96 detection points of each probe head is further normalized to account for any instrument effects.

#### 2.3.1. Subtraction Technique

In all experimental cases, a subtraction technique is employed to remove the background noise and improve the optical contrast between the target(s) and the background. The technique is implemented differently for reflectance (simultaneous bilateral) and transillumination based experimental studies. In the case of reflectance studies, two identical cubical phantoms are employed during each experimental study, one with and the other without a target. Subtraction technique is implemented by subtracting the normalized average intensity data (*i.e.*, corresponding to the 96 detection points of a given probe head) obtained from the phantom without a target (*i.e.*, background image) w.r.t the data from the phantom with a target (*i.e.*, target + background image). In the case of transillumination studies, a single probe head of the forked design is used during the studies. Here, a phantom with a target is initially imaged, followed by imaging the same phantom (at the same location) without the target. Subtraction of the normalized average intensity data from the phantom without the target w.r.t the phantom with the target yields a subtracted image with an improved optical contrast.

#### 2.3.2. Remove Detectors around Sources

Despite applying the subtraction technique, artifacts existed typically at the detection points close to the points of illumination, say ∼0.5 cm. Hence to remove any residual excitation leakage of the sources close to the detection points, the detection points located 0.5 cm around the points of illumination were removed. Removing these detection points tends to minimize the artifacts due to excitation leakage.

#### 2.3.3. Polynomial Fitting

After applying subtraction technique and removal of detectors around sources, the discrete intensity data across the detector points of a probe head surface are plotted as surface contour plots for a clear visual representation of the images. Plotting of these remaining sparse detection points’ subtracted intensity data (spaced 0.5 cm w.r.t each other) as surface contour plots via linear interpolations (as built-in Matlab functions) displayed multiple artifacts with no distinct target. Hence higher order polynomial fitting interpolations were attempted to better represent the 2D surface images. From extensive simulation studies involving phantoms with single targets, it was observed that the 5th degree polynomial was the best fit to the detection points (at similar locations as in experimental cases). Hence, a double regression algorithm using the 5th degree polynomial fit was developed and implemented to the experimental data points (after applying all the post-processing techniques described in Sections 2.3.1 and 2.3.2), in order to generate 2D surface contour images with minimal artifacts. At this stage, the developed polynomial fitting approach is limited to single target based phantom data and not for multiple targets.

#### 2.3.4. Target Location and Size

The centroid of the detected 2D target location was estimated from the maximum intensity detected in the 2D surface contour plot (obtained via polynomial fit). Full-width half max (FWHM) values were calculated in both x- and y-axis of the polynomial fitted experimental data and further averaged to estimate the 2D target size (as diameter). The measurement errors for each experimental case were evaluated from the average standard deviation across the 10 repeated measurements of normalized intensity data across all the detector points. The recovered or detected T:B contrast ratio was also evaluated as the ratio of the maximum detected intensity in the true target region w.r.t. the mean background intensity.

## 3. Results and Discussion

### 3.1. Effect of Data Post-Processing on Target Detection

**Figure 3.**
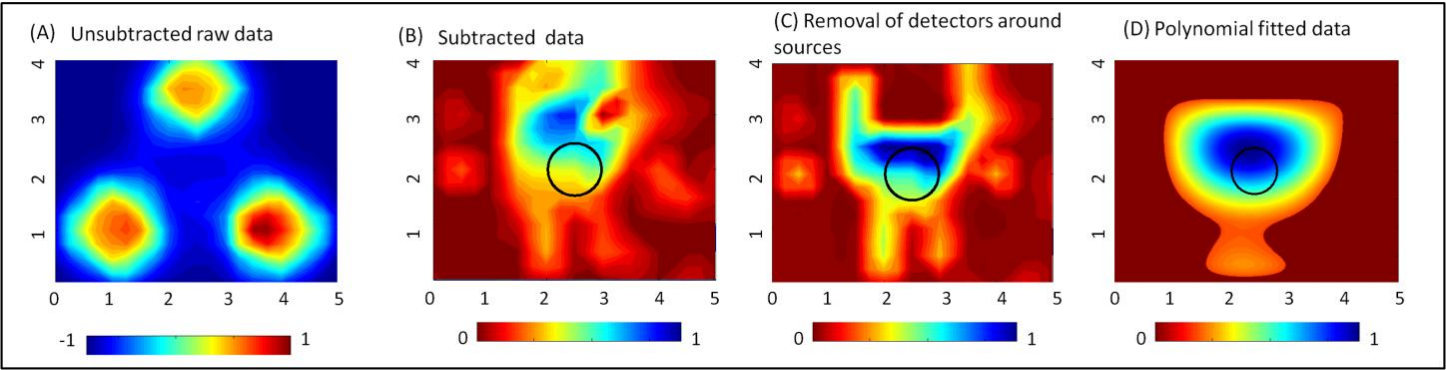
Two-dimensional surface contour plots of the experimental data at various stages of the implemented post-processing techniques: (**A**) unsubtracted raw data, (**B**) subtracted data, (**C**) data after removal of detectors around the sources, and (**D**) 5th degree polynomial fitted data. The sample data shown here is for experimental reflectance data using a 0.45 cc target located 1.5 cm deep.

The effect of implementing the proposed data post-processing techniques (described in Section 2.3) on actual experimental data is shown in Figure 3 for a reflectance imaging case. The data is normalized at each stage of the data post-processing technique applied. For a 0.45 cc target located 1.5 cm deep from the tissue surface, the 2D surface map of the detected (unsubtracted) intensity does not differentiate the target from the background. In fact, there is a strong reflected intensity *i.e.*, excitation leakage at the detectors located around the 3 point sources (see Figure 3(A)). Upon subtracting a background image (obtained from a phantom with no target), the effect of the strong excitation leakage is significantly reduced, as shown in the subtracted image in Figure 3(B). The target is detectable (at [2.53, 3.08] cm) in the subtracted image, but appears to be shifted from its true location, [2.5, 2] cm by 1.08 cm. Further removal of all detectors that immediately surround the source allowed for the detected target to locate closer ([2.49, 2.23] cm) to its true location (see Figure 3(C)) with a 0.23 cm distance-off. The 2D surface contour plots for Figure 3(A–C) are based on the Matlab’s linear interpolated contour plots. Upon applying the developed 5th degree polynomial fit to the post-processed 2D experimental (discrete) data points, the target localization is distinct with no artifacts (see Figure 3(D)). From these results, it is obvious that post-processing of the experimental optical data plays a significant role in improved target differentiation from the background with minimal artifacts. The proposed polynomial fitting approach is limited to tissue phantoms with a single target and not multiple targets. Currently, extensive simulation studies are carried out to develop appropriate techniques to better represent contour plots of tissue phantom surfaces with multiple targets as well.

### 3.2. Reflectance Studies

**Figure 4.**
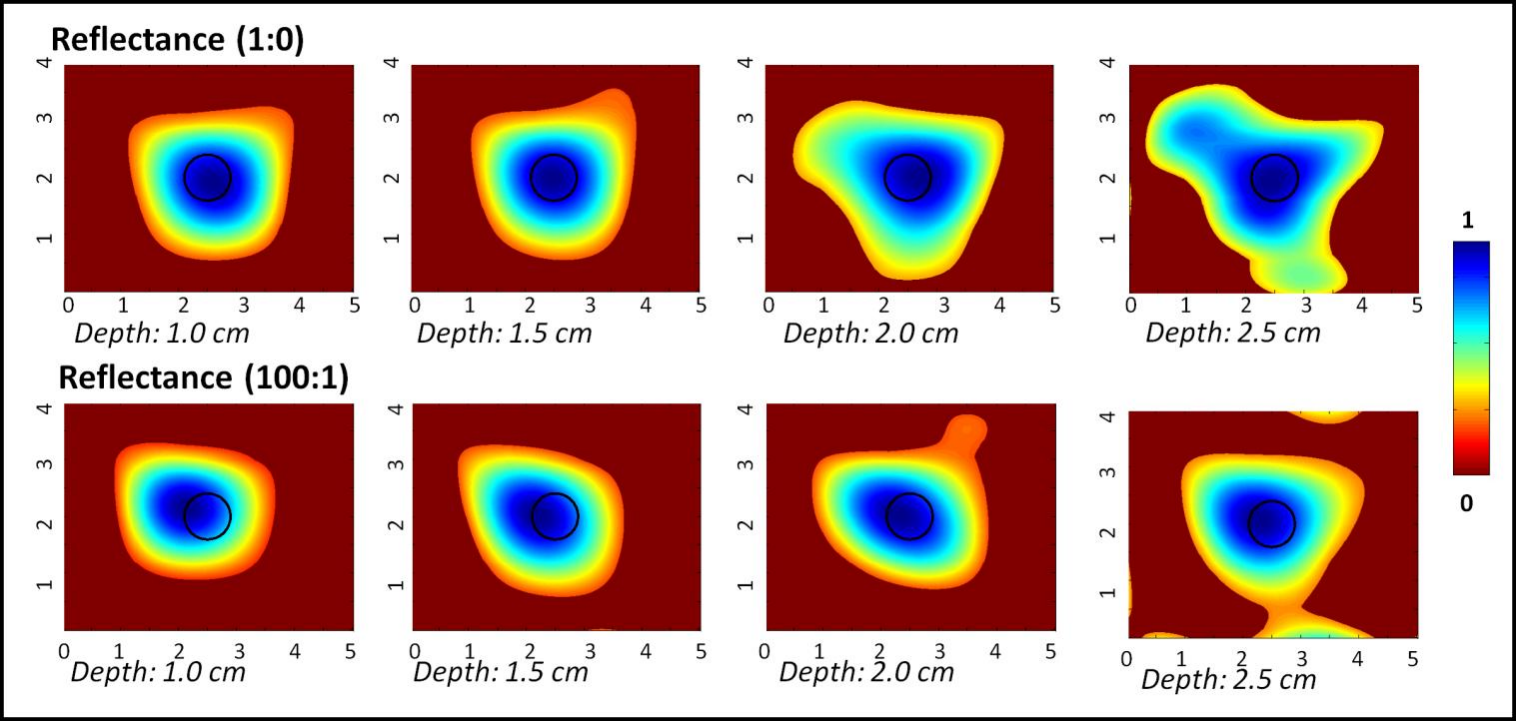
Two-dimensional surface contour plots (via 5th degree polynomial fit) of reflectance imaging studies of a 0.45 cc spherical target at 1:0 and 100:1 T:B India Ink contrast, located at various depths. The black hollow circle represents the true 2D target location.

Figure 4 shows the 2D surface contour plots (via polynomial fit) of the reflected (and subtracted) intensity signals obtained from a 0.45 cc target placed at various depths across the 10 × 10 × 10 cm^3^ phantom. Each experimental case was normalized since the instrumentation settings were different for each depth and T:B contrast ratio cases. From the plots, it is obvious that the target was detectable up to 2.5 cm deep, at both the chosen T:B (India Ink) contrast ratios. At depths of 3 cm and higher, the targets were not detectable in simulation studies and hence not imaged. The measurement errors were consistently ∼0.2 across any given target depth (up to 2.5 cm). The target was localized closer to its true location in all cases. The detected target location, target size, measurement errors and T:B detected contrast are given in Table 2.

### 3.3. Transillumination Studies

**Figure 5.**
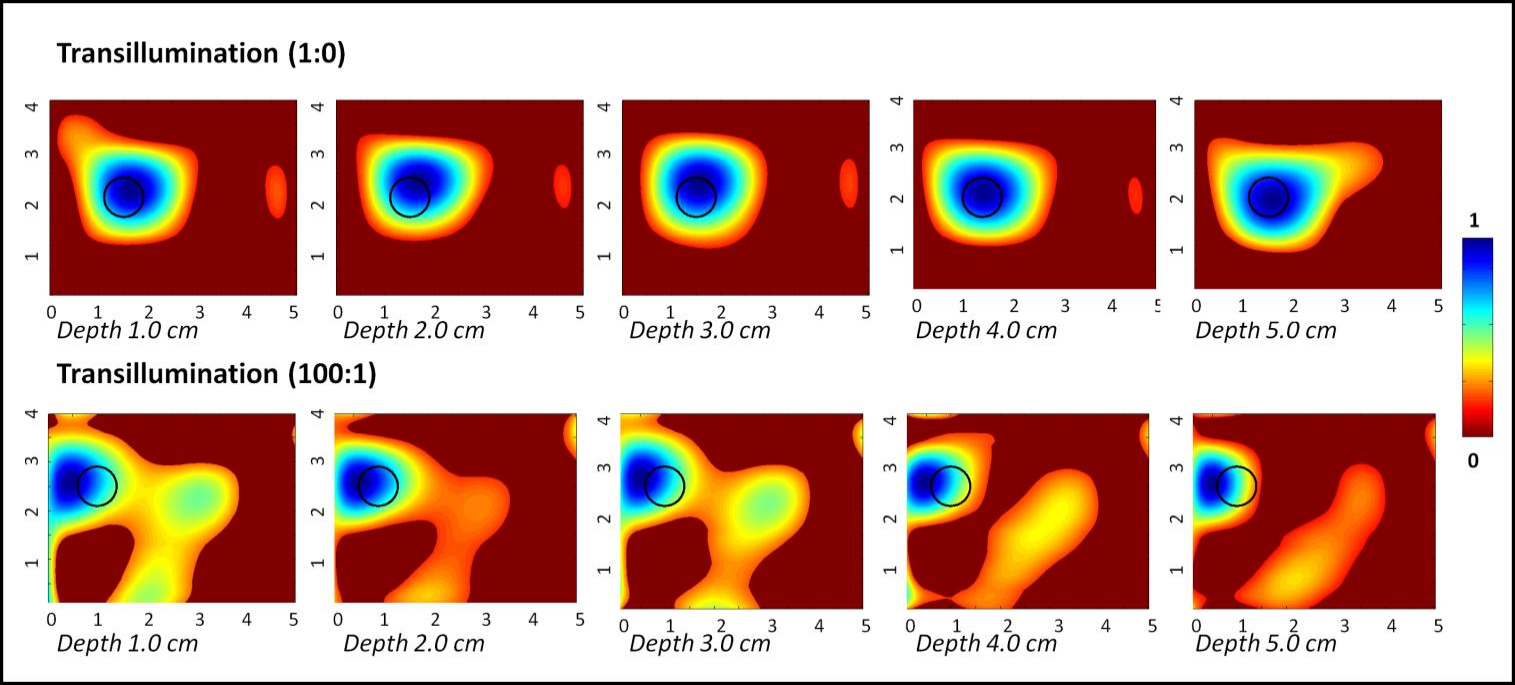
Two-dimensional surface contour plots (via 5th degree polynomial fit) of transilluminated reflectance imaging studies of a 0.45 cc spherical target at 1:0 and 100:1 T:B India Ink contrast, located at various depths. The black hollow circle represents the true 2D target location.

Figure 5 shows the 2D surface contour plots (via polynomial fit) of the transilluminated (and subtracted) intensity signals obtained from a 0.45 cc target placed at various depths across the 10 × 10 × 10 cm^3^ phantom. From the plots, it is obvious that the target was detectable across the entire depth of the phantom (*i.e.*, 5 cm deep), at both the chosen optical contrast ratios. The target was localized closer to its true location in all cases (≤0.5 cm in most cases), although there were obvious artifacts without the data post-processing techniques. The measurement errors were two orders of magnitude smaller for 1:0 contrast cases in comparison to the 100:1 contrast cases. The detected target location, target size, measurement errors and T:B detected contrast are given in Table 3. The measurement error was significantly smaller for 1:0 contrast cases of transillumination studies compared to reflectance imaging studies, and hence reported up to 4 significant digits.

The progression of images exhibit that as the target is closer to the detection surface (*i.e.*, further away from the illumination surface), where the optical detection fibers are placed a reduction in artifacts is visible, as well as an improvement in location estimation. Transillumination measurements in general provide greater depth information of the tissues over reflectance measurements. Typically most studies have focused on using single point source illumination to demonstrate the effectiveness of transillumination measurements. The current studies are different in terms of the source illumination configuration. Unlike the typically used sequential point illumination employed by other researchers, the Gen-2 hand-held imager can perform simultaneous multiple point illumination towards enhanced imaging rates. Thus, even simultaneous illumination, which can have null (intensity) points from interferences, can still detect deep targets based via transillumination imaging over reflectance, independent of the illumination configuration.

### 3.4. Preliminary *in-Vivo* Reflectance Breast Imaging

**Figure 6.**
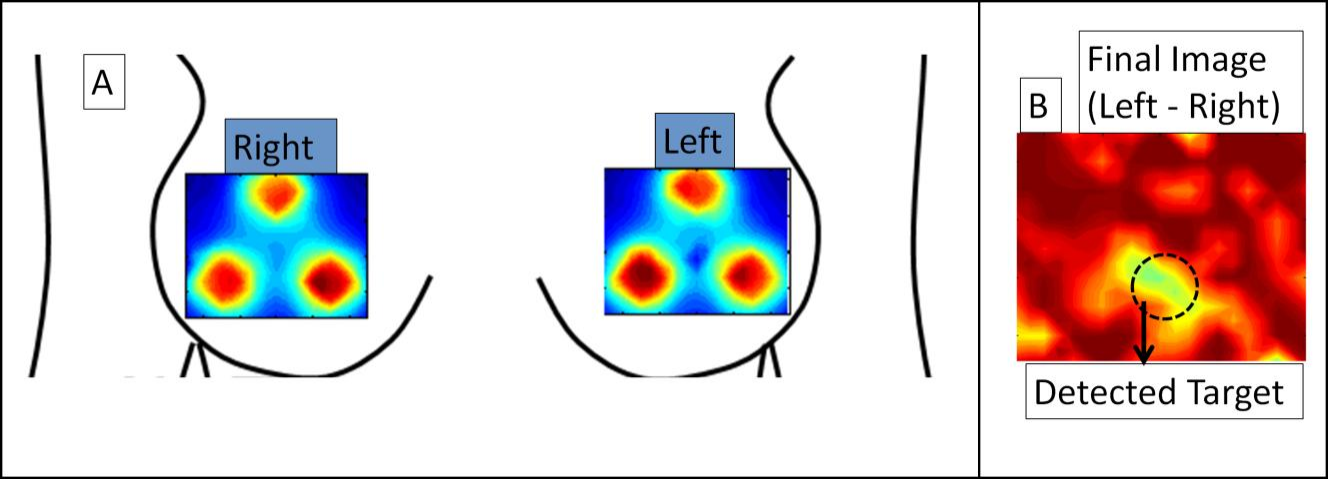
Simultaneous bilateral imaging using Gen-2 hand-held imager showing (**a**) 2D surface contour plot of intensity distribution on left and right breast (**b**) final subtracted image of left-right breast, in a normal breast with a 0.45 cc absorption target embedded under left breast flap at 6’o clock position. Final image in (b) differentiates target distinctly from background artifacts, with max absorption (in yellow) at target site.

Preliminary *in-vivo* breast imaging study was performed on a normal human subject with a 0.45 cc absorption target (India Ink) placed in the inter-mammography fold of the left breast (at 6 ’0 clock position). CW-based simultaneous bilateral reflectance imaging was performed on both the breasts at symmetric locations. While the unsubtracted images of left and right breasts did not detect the tumors, the subtracted image (left-right) distinctly differentiates the target (even prior to applying other data post-processing techniques described in Section 2.3), as shown in Figure 6. This preliminary study establishes the capability of the Gen-2 imager to detect targets *in-vivo* human breast tissues via simultaneous bilateral imaging. Extensive clinical studies are currently carried out to determine the performance of the Gen-2 imager towards both reflectance and transillumination imaging on normal and breast cancer subjects.

## 4. Conclusions

A Gen-2 hand-held optical imager with a unique forked hand-held probe design was developed towards both reflectance and transillumination imaging. In this study, the feasibility of reflectance and transillumination diffuse optical imaging was demonstrated on tissue phantoms. During reflectance imaging targets up to 2.5 cm deep were localized from 2D surface images. During transillumination imaging, targets up to 5 cm deep were localized. It can be observed from these experimental cases that transillumination provides a greater target depth recovery than reflectance imaging. It was also observed that transilluminated signals get weaker when the target is further away from the detected surface (or closer to the illumination surface), especially as T:B contrast ratio reduces.

The unique capability of the Gen-2 hand-held optical imager to perform both reflectance and transillumination imaging is an advantage. For instance a target closer to a surface can be detected by reflectance even at lower T:B contrast ratios, at places where transillumination can be limited by tissue volume or depth. Also flexibility to illuminate one or more point sources simultaneously allows for enhance imaging rates while still detecting deeper targets. Currently, *in-vivo* studies are ongoing on normal and breast cancer subjects using the Gen-2 hand-held optical imager. In a clinical environment, transilluminated optical images can be compared to the gold-standard X-ray mammography images using a portable hand-held imager.

On a different perspective, in over 75% of the breast cancer cases, tumors are unilateral (*i.e.*, in a single breast) before it sometimes spreads to the other breast tissue and/or elsewhere. By performing bilateral imaging of both the breast tissues simultaneously, where only one of the breasts is diseased, we can better differentiate the diseased *vs*. normal tissue with minimal instrument artifacts and improved T:B contrast. The hand-held optical imagers to date are neither capable of simultaneous imaging of both the breast tissues, nor single breast tissue imaging over larger areas, unlike the Gen-2 hand-held imager. Additionally, the Gen-2 imager’s capability to perform (sequential or simultaneous) bilateral reflectance imaging during clinical breast cancer imaging allows its direct comparison to the reflected ultrasound images. The added advantage of optical imaging is that it is a functional imaging approach, which measures the changes in blood flow or physiological changes in terms of HbO, HbR *etc.*, unlike the anatomical imaging approach (e.g., x-ray and ultrasound). This allows potential early stage tumor diagnosis, especially upon using external fluorescent contrast agents. Currently, work is carried out to register the positional information of the Gen-2 hand-held probe during imaging [30], such that 3D tomographic imaging can also be performed.

## Figures and Tables

**Table 1.** Experimental cases for simultaneous bilateral reflectance and transillumination imaging studies. In all the studies 3% Liposyn solution was used in the background phantoms and a 0.45 cc target was with 0.08% India Ink + 3% Liposyn solution. In 100:1 contrast ratios, a 0.0008% India Ink is added to the background.

Imaging Approach	T:B India Ink contrast	Target depth (cm)
Reflectance	1:0	(1.0),(1.5),(2.0),(2.5)
	100:1	(1.0),(1.5),(2.0),(2.5)
Transillumination	1:0	(1.0),(2.0),(3.0),(4.0),(5.0)
	100:1	(1.0),(2.0),(3.0),(4.0),(5.0)

**Table 2.** Quantitative details of the detected target location, size, recovered T:B contrast and measurement errors for different experimental cases using the Gen-2 imager towards reflectance imaging studies.

Contrast ratio (India Ink)	True target location [x,y] (cm)	True target depth, z (cm)	Detected Location [x,y] (cm)	Distance off from true [x,y] location (cm)	Recovered T:B contrast	Estimated target diameter (cm)	Measurement error
1:0	[2.50,2.00]	1.00	[2.59,1.92]	0.120	8.30	1.88	0.22
		1.50	[2.47,2.00]	0.030	7.40	1.94	0.22
		2.00	[2.70,2.02]	0.200	4.30	0.78	0.22
		2.50	[2.46,1.90]	0.110	12.5	0.94	0.22
100:1	[2.50,2.00]	1.00	[2.12,2.16]	0.410	8.80	1.53	0.21
		1.50	[2.14,2.15]	0.390	8.20	1.62	0.21
		2.00	[2.15,2.14]	0.380	9.30	1.54	0.21
		2.50	[2.27,2.08]	0.240	5.00	1.62	0.22

**Table 3.** Quantitative details of the detected target location, size, recovered T:B contrast and measurement errors for different experimental cases using the Gen-2 imager towards transillumination imaging studies.

Contrast ratio (India Ink)	True target location [x,y] (cm)	True target depth, z (cm)	Detected Location [x,y] (cm)	Distance off from true [x,y] location (cm)	Recovered T:B contrast	Estimated target diameter (cm)	Measurement error (×100%)
1:0	[1.50,2.00]	1.00	[1.58,2.40]	0.410	19.3	1.70	0.001
		2.00	[1.63,2.43]	0.450	19.3	0.86	<0.001
		3.00	[1.59,2.44]	0.450	17.8	0.96	0.001
		4.00	[1.55,2.35]	0.350	17.7	0.88	0.001
		5.00	[1.65,2.04]	0.150	19.7	0.42	0.002
100:1	[1.00,2.50]	1.00	[0.55,2.82]	0.550	3.86	1.24	0.252
		2.00	[0.63,2.79]	0.470	4.38	1.14	0.245
		3.00	[0.61,2.82]	0.500	3.95	0.95	0.241
		4.00	[0.60,2.85]	0.530	4.265	0.97	0.248
		5.00	[0.57,2.74]	0.490	5.756	0.74	0.241
